# Antimicrobial Activities and Biofilm Inhibition Properties of *Trigonella foenumgraecum* Methanol Extracts against Multidrug-Resistant *Staphylococcus aureus* and *Escherichia coli*

**DOI:** 10.3390/life13030703

**Published:** 2023-03-05

**Authors:** Rawaf Alenazy

**Affiliations:** Department of Medical Laboratory, College of Applied Medical Sciences-Shaqra, Shaqra University, Shaqra 11961, Saudi Arabia; ralenazy@su.edu.sa

**Keywords:** multidrug resistance, bacterial infections, antibiotics, natural compounds, *T. foenumgraecum*, *Staphylococcus aureus*, *Escherichia coli*, gas chromatography-mass spectrometry (GC-MS) analysis, phytochemical analysis

## Abstract

Multidrug-resistant bacteria are becoming the leading cause of death globally due to their resistance to many currently used antibiotics. Bacteria naturally have intrinsic resistance or acquired resistance to certain commonly used antibiotics. Therefore, searching for novel compounds has become necessary. *Trigonella foenumgraecum* extract was evaluated for antimicrobial and antibiofilm activities against multidrug-resistant bacteria *Staphylococcus aureus* and *Escherichia coli*. The minimum inhibitory concentration and minimum bactericidal concentration of the extract were also determined. Moreover, gas chromatography-mass spectrometry (GC-MS) analysis was used to identify the phytochemical components present in the extract. GC-MS analysis revealed that *T. foenumgraecum* extract contains major compounds such as Phenol, 2-methoxy-3-(2-propenyl)-, n-Hexadecanoic acid, and 9,12,15-Octadecatrienoic acid. Both bacterial strains showed resistance to some of the antibiotics tested. *T. foenumgraecum* showed inhibitory activity against the tested bacterial strains with a MIC of 500 µg/mL and MBC of 1000 µg/mL. The methanol extract decreased the biofilm activity of both *E. coli* and *S. aureus* below the sub-minimum inhibitory concentration. The extract showed antibacterial and antibiofilm activity against the tested bacterial pathogens.

## 1. Introduction

Multidrug-resistant bacteria infections are becoming a significant threat to public health [1]. The main reason for the increase in the rate of resistance in these strains is the excessive and unsystematic usage of antibiotics, which have lost their effectiveness due to bacterial resistance [2]. The multidrug-resistant bacteria issues are prevalent in many aspects of life and are not limited to hospitals or health care, but also include important aspects such as food and the environment [3]. In hospitals, infections of *Staphylococci* and *E. coli* are mostly reported as hospital-acquired infections [4]. In order to face the antimicrobial agents in the surrounding environment, most bacteria resort to forming a complex multicellular biofilm structure [5], which leads to further complication of the issue of antimicrobial resistance and limits the available treatment options. Multidrug-resistant bacteria have the ability to form biofilms which leads to preventing antimicrobials from permeating into bacterial cells which leads to increased bacterial growth, prolonged chronic infections, and tolerance of systemic host defenses and external stresses [6,7]. These properties are harmonized by pathogens in a partnership relation with a signaling mechanism called quorum sensing [7]. This is carried out by synthesizing and secreting extracellular signaling molecules. Staphylococcal accessory regulator A (SarA) is a quorum regulator and global accessory regulator (agr) with the capacity to control pathogenicity and biofilm formation in other species [8]. Biofilm is considered a significant marker for infection that confers resistance to antibiotics and disinfectants [9]. Thus, increased virulence of pathogens and infection control becomes difficult, leading to the search for novel treatment plans for multidrug-resistant bacteria. There are several natural products used to control pathogens and inhibit biofilm formation [10]. Phytochemicals are one of the important plant materials used widely in pharmaceuticals and food industries to control pathogens. These chemicals directly affect the membrane integrity and increase the permeability of membranes affecting the outflow of intracellular components and leading to the death of pathogens [11]. In addition, these chemicals could act as efflux pump inhibitors in pathogenic bacteria leading to inhibiting these pumps and increasing the accumulation of the antibiotics inside the cells, which leads to the death of the pathogens as well [12]. 

Among these, *T. foenumgraecum* (fenugreek), used as a spice in foods, is considered for its high medicinal and nutraceutical value. *T. foenumgraecum* is rich in lysine, L tryptophan, and fibers. Some alkaloids, sapogenins, flavonoids, and steroids were the major components; moreover, some volatile compounds such as anethole and Stallone were also reported [13] (Singh et al. 2020). It also contains fenugreeke, saponins, nicotinic acid, coumarin, sapogenins, phytic acids, scopoletin, and trigonelle that are reported for various biological effects. Moreover, these phytochemicals possess several effects such as anticancer, antidiabetic, antioxidant, and antimicrobial activities. The *T. foenumgraecum* was investigated in order to identify the main major phytochemical compounds that could be used based on their biological activities. The purpose of this study is to evaluate the antimicrobial activities and biofilm inhibition properties of phytochemicals of *T. foenumgraecum* on the multidrug-resistance of *S. aureus* and *E. coli*.

## 2. Materials and Methods

### 2.1. Materials

Bacterial isolates from clinical samples identified by morphological and biochemical characteristics were used in the present study. The cultures of *S. aureus* ATCC 25923 and *E. coli* ATCC 35218 were stored in a nutrient agar slant. The cultures were enriched using tryptic soy broth for the studies. For the preparation of the *T. foenumgraecum* (fenugreek) extract, 100 g of the powdered material was soaked in methanol (900 mL) for 72 h in a shaking incubator. The preparation was filtered through muslin cloth and the solvent was completely evaporated. The obtained extracts were weighed and used for further studies. 

### 2.2. GC-MS Analysis of T. foenumgraecum

The GC-MS 7890A and 5975C VL MSD (Agilent technologies, Santa Clara, CA, USA) systems were chosen to analyze the fenugreek extract. The extract (100 µL) was mixed with the water-ethyl-acetate mixture (250 µL) through forceful shaking to gather and concentrate the sample’s top layer. Additions of trimethylchlorosilane and trifluoroacetamide were made, followed by (BSTFA-99 µL + TMCS- µL), and finally, 10 µL of pyridine was added. The mixture was heated for 30 min at 60 °C and the samples were transferred to GC vials. The samples were dried using liquid nitrogen before being dissolved in methanol and analyzed by GCMS. The dried sample was dissolved in HPLC-grade methanol. The sample (1 µL) was injected using an Agilent capillary column (DB5MS) with dimensions of 30 mm, 0.25 mm internal diameter, and a film thickness of 0.25 microns. A temperature of 270 °C and a pressure of 80 kPa was maintained in the injector. Using helium as a carrier gas, the GC procedure was completed in 25 min. The NIST mass spectral database and the collected mass spectra were used to identify the compounds.

### 2.3. Antibiotic Resistance

The bacteria were subjected to antibiotic sensitivity tests for the determination of multidrug-resistance using the conventional antibiotics used for the treatment of infections. Amoxicillin (25 mcg), bacitracin (10 mcg), chloramphenicol (30 mcg), ciprofloxacin (5 mcg), erythromycin (15 mcg), gentamicin (10 mcg), and tetracycline (30mcg) discs were used in the study, using the Kirby–Bauer disc diffusion method. Bacterial cultures with 0.5 McFarland’s standard turbidity were swabbed on Mueller–Hinton agar plates with sterile cotton swabs. Antibiotic discs were placed and the plates were incubated at 37 °C. The inhibition zones were measured by using a millimeter scale and the results were analyzed using reference tables provided by the Clinical and Laboratory Standards Institute (CLSI), standards, 2016.

### 2.4. Antibacterial Activity of the Extracts

The agar well diffusion method was used to determine the antibacterial activity of extracts [14]. Bacterial cultures were grown on nutrient broth at 37 °C and diluted to get 0.5 McFarland standard turbidity. These cultures were swabbed on Mueller–Hinton agar using a sterile cotton swab. Wells were made on agar by using a cork borer and the bottoms of the wells were sealed with a few drops of Mueller–Hinton agar. The wells were filled with different concentrations of extracts and incubated at 37 °C for 24 h. The zone of inhibition was measured and recorded.

#### 2.4.1. Minimum Inhibitory Concentration and Minimum Bactericidal Concentration

The lowest concentration of the extract that inhibits the visible growth of bacteria was calculated as the minimum inhibitory concentration (MIC). For the determination of MIC, the cell density was measured at 600 nm with a control media with extract as blank. The minimum bactericidal concentration (MBC) was determined as the minimum inhibitory concentration of the extracts that inhibited the viability of the cells up to 99.9%. For MBC, the broth was plated in nutrient agar after dilution (up to 10^8^ fold) to check the viability of cells. Untreated broth with bacterial culture was taken as control. These experiments were carried out twice independently.

#### 2.4.2. Antibiotic Phytochemical Inhibition Assay

The dual combination of antibiotic and phytochemical extracts was carried out to determine the efflux pump inhibition by the extracts using the modified Kirby–Bauer method [15]. Briefly, Mueller–Hinton agar was prepared, sterilized, and incorporated with different concentrations of phytochemicals and poured into sterile Petri dishes. Overnight grown cultures with turbidity matching with McFarland solution (0.5) were swabbed on the medium with sterile cotton swabs. Antibiotic discs such as ciprofloxacin, erythromycin, and tetracycline were placed to determine the efflux pump inhibition properties of the extract. The plates were incubated at 37 °C for 24 h. The inhibition zone diameter was measured and analyzed according to the guidelines of CLSI (Clinical and Laboratory Standards Institute (CLSI) Performance Standards for Antimicrobial Susceptibility Testing; Wayne, PA, USA, 2005). The experiment was performed in triplicates and the mean inhibition zone diameter was noted.

### 2.5. Antibiofilm Activity Spectrophotometric Assay

The ability of the extracts to prevent biofilm formation was determined. The bacterial cultures (10^8^ CFU/mL) were inoculated in a microtiter plate along with extracts with MIC concentration. The plates were incubated at 37 °C for 24 h and the formed biofilm was measured using crystal violet. For control, the wells were inoculated with culture and sterile distilled water. The biofilm inhibition was calculated based on the formula.

% of inhibition = 100 − (OD_570_ sample/OD_570_ control) × 100

### 2.6. Antibiofilm Activity Microscopic Assay

For detecting the Antibiofilm activity of the extracts, a coverslip assay was performed. Bacterial cultures were inoculated in tryptic soy broth and the sterile coverslips (UV sterilized) were placed in the test tubes to attain a 90° angle relative to the bottom of the tube. Different concentrations (125, 250, 500 µg/mL) of the extracts were added to the medium. The meniscus of the medium reached up to the center of the coverslip and was incubated at 37 °C for 24 h. The coverslips were removed and washed with cold tap water to remove the non-adherent cells. Coverslips were stained by Gram staining by immersing the coverslips in different stains and then being air dried. The biofilm formed on the surface of the coverslips was observed by using a light microscope [16]. Control tubes were inoculated with bacterial cultures without extracts.

### 2.7. Minimum Biofilm Inhibitory Concentration

For determination of minimum biofilm inhibitory concentration, the bacterial cultures (100 µL) were inoculated along with different concentrations (1–1000 µg/mL) of extract in polystyrene microtiter plates for 24 h. The cells were removed by washing them with phosphate buffer solution three times. The remaining cells were fixed with 99% methanol. The fixed cells were stained with 0.2% crystal violet (150 µL) for 20 min. The excess stains were removed and slowly washed with cold tap water and dried at room temperature. The cell-bound crystal violet was eluted by using 33% acetic acid and the optical density was recorded at 595 nm. Minimum biofilm inhibitory concentration was the lowest concentration of the extract that inhibits 50% biofilm formation compared with the culture without extracts as MBIC_50_ and inhibition at 90% is noted as MBIC90 [17]. 

## 3. Results

**Figure 1:**
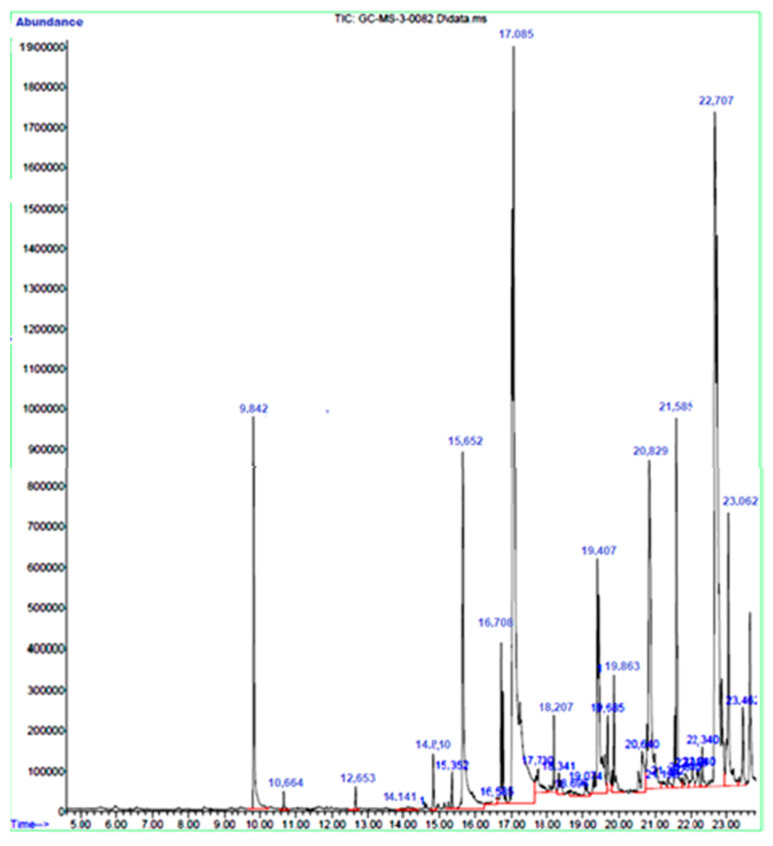
GCMS chromatogram of fenugreek extract.

The major components that were identified in the extract were Phenol, 2-methoxy-3-(2-propenyl)-, n-Hexadecanoic acid, 9,12,15-Octadecatrienoic acid, Butyl 9,12-octadecadienoate, 9,12-Octadecadienoic acid (Z, Z)-, 3H,6H-Thieno [3,4-c] isoxazole, E, Z-1,3,12-Nonadecatriene, and gamma. –Tocopherol (Table 1). Some components were identified in smaller amounts which may be responsible for the antibacterial and antibiofilm activity of the extract. The extracts’ vast majority of chemicals were also detected using this method (Figure 1).

The antibiotic sensitivity pattern showed by *E. coli* and *S. aureus* was studied. *E. coli* showed resistance or an intermediate pattern towards antibiotics such as erythromycin, ciprofloxacin, and amoxicillin. This strain was sensitive to most of the antibiotics tested. *S. aureus* showed a sensitive pattern to most of the antibiotics except gentamycin and erythromycin.

The antibiotic potencies of *T. foenumgraecum* on *S. aureus* and *E. coli* were studied (Table 2). The methanol extract showed the least antibacterial activity at 200 µg/mL against the pathogens and the increasing concentration of the extract showed maximum activity at 800 (µg/mL) against both bacterial strains.

The minimum inhibitory concentration and minimum bactericidal concentration of *T. foenumgraecum* methanol extracts on *S. aureus* and *E. coli* were studied (Table 3). Turbidity was observed in the tubes with *S. aureus* and *E. coli* at a concentration of 250 µg/mL. There was no visible turbidity at 500 µg/mL with both *S. aureus* and *E. coli* indicating the minimum inhibitory concentration. Hence, these concentrations were tested for minimum bactericidal concentration.

**Figure 2:**
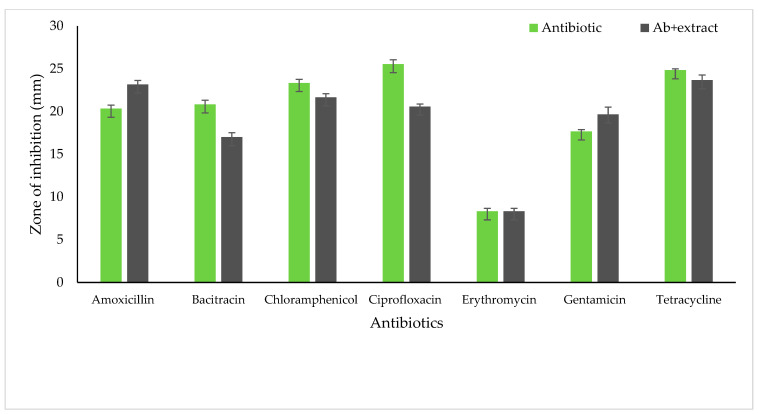
Antibiotic phytochemical inhibition assay on *S. aureus* (inhibition zone is shown in mm).

**Figure 3:**
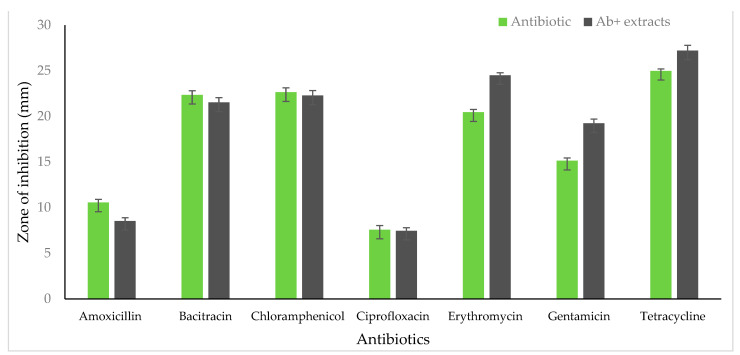
Antibiotic phytochemical inhibition assay on *E. coli.* (Inhibition zone is shown in mm).

The synergetic activity of the extracts along with the antibiotics was tested by disc diffusion assay. The bacterial isolates showed resistance to some of the antibiotics tested. The inhibition zone of *S. aureus* increased the diameter with amoxicillin and gentamycin (Figure 2). However, the extracts in combination with antibiotics did not have any effect with bacitracin, chloramphenicol, ciprofloxacin erythromycin, and tetracycline on *S. aureus*. In *E. coli*, antibiotics showed an increase in the inhibition zone with erythromycin, gentamycin, and tetracycline when tested along with the extracts (Figure 3). Antibiotics such as amoxicillin, chloramphenicol, ciprofloxacin, and bacitracin did not show a synergetic effect in inhibiting growth.

The methanol extract decreased the biofilm activity of both *E. coli* and *S. aureus* below the minimum inhibitory concentration. Biofilm formation in *E. coli* was inhibited (48.2%) at 250 µg/mL and 85.3% inhibitory activity was observed with a higher concentration (500 µg/mL) of the extract. Similarly, the biofilm formation in *S. aureus* was inhibited at a sub-MIC of the extract with a reduction of 70.95% at 500 µg/mL concentration.

**Figure 4:**
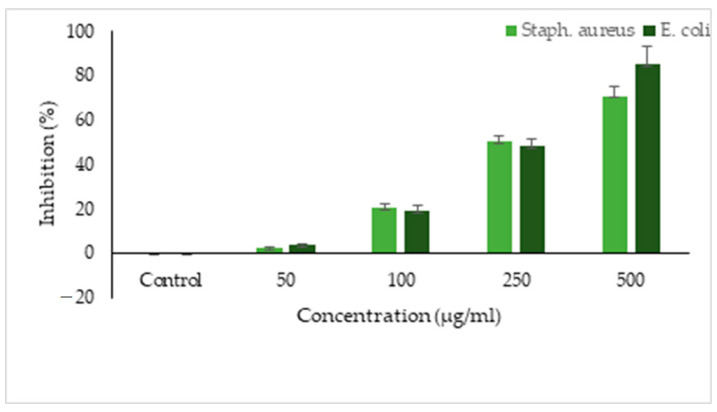
Antibiofilm inhibition activity of *T. foenumgraecum* methanol extracts on *S. aureus* and *E. coli*.

**Figure 5:**
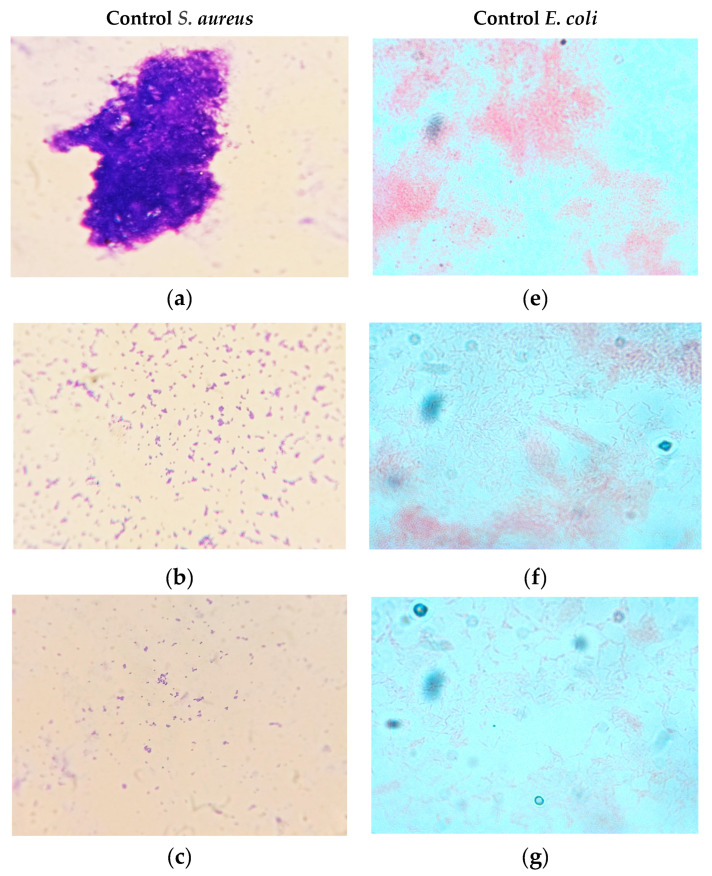

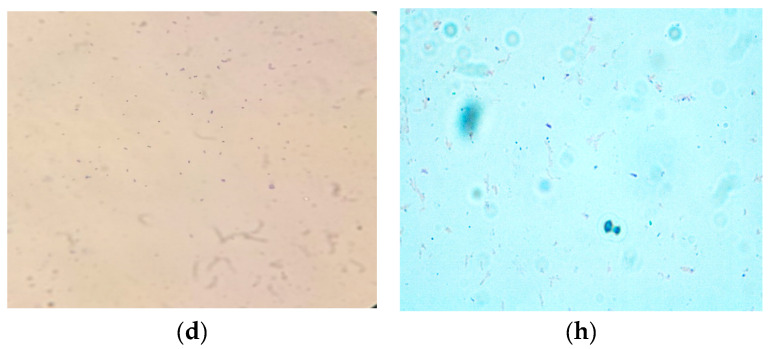
Antibiofilm activity by coverslip assay (Microscopic images) of *T. foenumgraecum* methanol extracts ((**a**) control; (**b**) 125 µg/mL; (**c**) 250 µg/mL; (**d**) 500 µg/mL) on *S. aureus* and ((**e**) control; (**f**) 125 µg/mL; (**g**) 250 µg/mL; (**h**) 500 µg/mL) on *E. coli*.

The disintegrated biofilm architecture in the coverslip and the number of increased planktonic cells, when compared with the control, show the inhibitory effect on biofilm formation (Figure 4). Light microscopic observations also confirmed that the higher the concentration of *T. foenumgraecum,* the greater the inhibition of bacterial growth, and the lower the concentration, the lower the inhibition (Figure 5). The reduced number of microcolonies on the coverslip in the presence of extracts might have resulted in the weak form of biofilm through a reduction in communication and surface adhesion.

**Figure 6:**
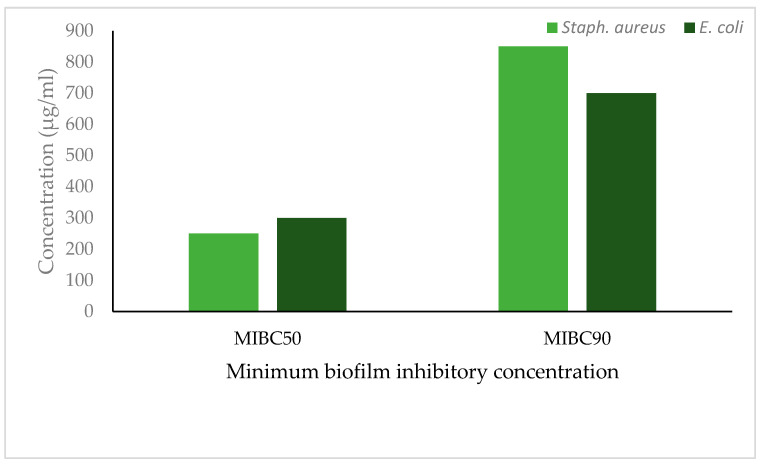
Minimum biofilm inhibitory concentration of *T. foenumgraecum* methanol extracts on *S. aureus* and *E. coli*.

*T. foenumgraecum* methanol extract efficiently inhibited biofilm formation and the minimum concentration to inhibit the biofilm formation in *S. aureus* was 250 µg/mL (MIBC50) and 850 µg/mL (MIBC90). In *E. coli*, the minimum concentration to inhibit biofilm was 300 µg/mL (MIBC50) and 700 µg/mL (MIBC90) (Figure 6). However, a lower concentration of the extract inhibited 50% of the biofilm in *S. aureus* and a higher concentration was required to inhibit 90% of biofilm formation.

## 4. Discussion

Fenugreek has been reported for many biological activities including antibacterial, anticancer, and anti-inflammatory around Asian and European countries [18]. Several biological active components and phytochemicals have been identified in fenugreek for their medicinal importance. Analysis using GC-MS enabled the identification of several components in the extract. Evaporation occurs above 270 °C for the majority of biochemical components present in the extracts. Since fenugreek contains lipid components and organic volatile ingredients, they were identified by GC-MS. The resistance pattern shown by these isolates to the commonly used antibiotics is due to the frequent and indiscriminate use in treatment [19]. *E. coli* is susceptible to all clinically used antibiotics, however, these bacteria have the capacity to accumulate resistant genes from other bacterial strains through horizontal gene transfer [20]. *S. aureus* is responsible for many types of infections and the increased usage of antibiotics has led to the development of resistance to commonly used antibiotics. The resistance mechanism in *S. aureus* is a complex process and thereby, understanding the mechanism and treatment of infections of these antibiotic-resistant bacteria has become more difficult [21].

The antimicrobial potency of *T. foenumgraecum* has been reported in many clinical isolates [22]. However, the extract showed antibacterial activity against multidrug-resistant isolates of *S. aureus* and *E. coli*. Infections caused by these organisms in the immunocompromised host becomes serious and difficult to treat due to the development of resistance. For this reason, development of bactericidal phytochemicals from plant-based sources is receiving interest [18]. In the present study, the methanol extract showed significant activity against both Gram-negative and Gram-positive bacterial strains. Seeds of *T. foenumgraecum* have been reported for bioactive components including alkaloids such as trigonelline and choline, as well as polyphenols and saponins such as diosgenin, gitogenin, homorientin, neogitogenin, neogigogenin, saponaretin, etc. [23]. The presence of less polymerized free phenols and scopoletin (coumarin) that interrupt the electron transport chain of prokaryotes, polyphenols, and flavanoids are responsible for the antimicrobial activity of the extract [24].

The bacterial multiplication and growth were inhibited at 1000 µg/mL concentration. However, a low concentration of 500 µg/mL was inhibiting the visible growth and a higher concentration was required for complete inhibition of bacterial growth. Phytochemicals are beneficial and safe, and contain novel molecules that can inhibit the growth of pathogenic bacteria (Singh et al., 2020). Earlier studies on an ethanol extract of *T. foenumgraecum* showed inhibitory activities in bacterial isolates and the minimum inhibitory concentration ranged from 50-500 µg/mL of the extracts [18]. Phytochemicals in combination with antibiotics may interact with the pathways to inhibit the growth of the bacterial species thereby showing a synergetic effect. Further, some of these antibiotics lost their efficacy in combination with the methanol extract. The phytochemicals may have had an inhibitory or competitive inhibitory action on enzyme receptors to reduce the effect of antibiotics [25].

Extracts at lower concentrations had a decreased Antibiofilm activity. Acyl homoserine lactone-dependent quorum sensing plays a major role in biofilm formation in bacteria [26]. Methanol extract had a significant role in obstructing these molecules to inhibit the biofilm formation in both isolates. The presence of coumarin and flavonoids in the extracts inhibits biofilm formation [26]. The presence of an anti-quorum sensing compound in the culture medium may reduce population density as well as pathogenicity [27]. Methanol extract fraction of *T. foenumgraecum* has been reported for significant activity in interfering with quorum sensing and biofilm formation. A considerable reduction in biofilm formation without affecting microbial growth has been reported earlier on *Pseudomonas* and *Aeromonas* strains [28]. Fenugreek is a widely used medicinal plant that contains plenty of phytochemicals with flavor. It is also used as a spice. Several studies have been reported on the antibacterial activity of fenugreek ethanolic extract against *Staphylococcus aureus* and *Pseudomonas aeruginosa* in immunocompromised patients [18]. Even aqueous extract has been reported for antimicrobial activity [29]. In this study, the ability to control the bacterial pathogen through inhibiting biofilm formation has been reported.

## 5. Conclusions

*T. foenumgraecum* methanol extract showed antibacterial activity against the tested pathogens. It also inhibited biofilm formation in *Staphylococcus* and *E.coli*. Further research is needed for individual bioactive compounds present in *T. foenumgraecum* that are capable of inhibiting biofilm formation that may also serve as a novel, effective, and safe source of quorum-sensing inhibitory molecules.

## Figures and Tables

**Table 1:** The major components of fenugreek extract that were identified by GCMS.

Peak	Retention Time	Area (%)	Name of the Compound	Formula	RT Index
1.	9.842	4.29	Phenol, 2-methoxy-3-(2-propenyl)-	C_10_H_12_O_2_	1082.312
2.	10.664	0.20	Bicyclo [5.2.0] nonane, 2-methylene-	C_15_H_24_	1102.395
3.	12.653	0.21	4-Butylbenzoic acid,	C_11_H_14_O_2_	1154.181
4.	14.141	0.20	5,8-Epoxy-3H-2-benzopyran	C_11_H_14_O_2_	1187.849
5.	14.830	0.56	1,2-Benzenedicarboxylic acid	C_16_H_20_O_4_	1202.887
6.	15.352	0.40	Hexadecanoic acid, methyl ester	C_18_H_36_O_2_	1216.303
7.	15.652	6.81	n-Hexadecanoic acid	C_16_H_32_O_2_	1223.808
8.	16.585	0.32	2,3’-Bipyridine	C_10_H_8_N_2_	1246.262
9.	16.708	2.36	9,12-Octadecadienoic acid	C_18_H_32_O_2_	1249.128
10.	17.085	25.49	9,12,15-Octadecatrienoic acid	C_18_H_30_O_2_	1257.781
11.	17.730	1.05	1,6-Cyclodecadiene	C_10_H_16_	1272.152
12.	18.207	0.84	1,15-Pentadecanedioic acid	C_15_H_28_O_4_	1282.447
13.	18.341	0.51	1H-Tetrazole-1-ethanol, 5-amino-	CH_3_N_5_	1285.291
14.	18.696	0.33	2-Methyl-Z,Z-3,13-octadecadienol	C_19_H_36_O	1292.726
15.	19.074	0.38	Bicyclo [10.1.0]tridec-1-ene	C_13_H_22_	1300.619
16.	19.407	6.38	Butyl 9,12-octadecadienoate	C_22_H_40_O_2_	1309.122
17.	19.685	1.03	Hexadecanoic acid, 2-hydroxy-1-	C_19_H_38_O_4_	1316.111
18.	19.863	1.45	4,4’-Methylenebisphenol, 2,2’,6’	C_17_H_20_O_2_	1320.533
19.	20.640	1.50	Silane,methylenebis[dimethyl- silane	C_9_H_16_Si_2_	1339.387
20.	20.829	9.64	9,12-Octadecadienoic acid (Z,Z)-...	C_21_H_40_O_2_Si	1343.865
21.	21.196	0.25	1,4-Benzenediol, 2,5-bis(1,1-dim	C_16_H_26_O	1352.447
22.	21.352	0.22	Hexahydropyridine, 1-methyl-4-[4	C_13_H_19_NO_2_	1356.05
23.	21.585	4.83	3H,6H-Thieno [3,4-c]isoxazole	C_8_H_13_NOS	1361.382
24.	21.840	0.48	Benzenesulfonamide	C_6_H_7_NO_2_S	1367.153
25.	22.018	0.50	Eicosane	C_20_H_42_	1371.141
26.	22.240	0.44	1H-Indole, 5-methyl-2-phenyl-	C_15_H_13_N	1376.07
27.	22.340	0.51	2H-1-Benzopyran-6-ol, 3,4-dihydr	C_29_H_44_O_2_	1378.274
28.	22.707	23.49	E,Z-1,3,12-Nonadecatriene	C_19_H_34_	1386.28
29.	23.062	4.17	gamma.-Tocopherol	C_28_H_48_O_2_	1393.902
30.	23.462	1.17	beta.-Sitosterol acetate	C_31_H_52_O_2_	1402.912

**Table 2:** Antibiotic potencies of *T. foenumgraecum* on *S. aureus* and *E. coli*.

Potency (µg/mL)	*S. aureus*	*E. coli*
	Zone of Inhibition (mm)	
50	-	-
100	-	-
200	8	10
400	14	15
800	16	18

- No inhibition zone.

**Table 3:** Minimum inhibitory concentration and minimum bactericidal concentration of *T. foenumgraecum* methanol extracts on *S. aureus* and *E. coli*.

	Minimum Inhibitory Concentration (µg/mL)				Minimum Bactericidal Concentration (µg/mL)			
Bacterial isolates	125	250	500	1000	125	250	500	1000
*S. aureus*	-	-	+	+	-	-	-	++
*E. coli*	-	-	+	+	-	-	-	++

- indicates visible growth, + no visible growth, and ++ shows no observed growth when re-inoculated in media.

## Data Availability

Not applicable.
